# Quality of life improves early after gender reassignment surgery in transgender women

**DOI:** 10.1007/s00238-016-1252-0

**Published:** 2016-10-29

**Authors:** Ebba K. Lindqvist, Hannes Sigurjonsson, Caroline Möllermark, Johan Rinder, Filip Farnebo, T. Kalle Lundgren

**Affiliations:** 1 Clinic for Reconstructive Plastic Surgery, Karolinska University Hospital, 171 76 Stockholm, Sweden; 2 Dept. of Molecular Medicine and Surgery, Karolinska Institute, 171 77 Stockholm, Sweden; 3 Stockholm Craniofacial Center, Clinic for Reconstructive Plastic Surgery, A2:04, Karolinska University Hospital, 171 76 Stockholm, Sweden

**Keywords:** Gender reassignment surgery, Gender dysphoria, Quality of life, SF-36

## Abstract

**Background:**

Few studies have examined the long-term quality of life (QoL) of individuals with gender dysphoria, or how it is affected by treatment. Our aim was to examine the QoL of transgender women undergoing gender reassignment surgery (GRS).

**Methods:**

We performed a prospective cohort study on 190 patients undergoing male-to-female GRS at Karolinska University Hospital between 2003 and 2015. We used the Swedish version of the Short Form-36 Health Survey (SF-36), which measures QoL across eight domains. The questionnaire was distributed to patients pre-operatively, as well as 1, 3, and 5 years post-operatively. The results were compared between the different measure points, as well as between the study group and the general population.

**Results:**

On most dimensions of the SF-36 questionnaire, transgender women reported a lower QoL than the general population. The scores of SF-36 showed a non-significant trend to be lower 5 years post-GRS compared to pre-operatively, a decline consistent with that of the general population. Self-perceived health compared to 1 year previously rose in the first post-operative year, after which it declined.

**Conclusions:**

To our knowledge, this is the largest prospective study to follow a group of transgender patients with regards to QoL over continuous temporal measure points. Our results show that transgender women generally have a lower QoL compared to the general population. GRS leads to an improvement in general well-being as a trend but over the long-term, QoL decreases slightly in line with that of the comparison group.

Level of evidence: Level III, therapeutic study.

## Introduction

Gender dysphoria is a condition in which there is incongruence between the individual’s own perception of his/her sex and their biological phenotype [1]. There is a strong desire to undergo medical and surgical treatment to change the body in a way that is closer to one’s experienced gender in order to alleviate physical incongruence and gender dysphoria [2]. The prevalence of gender dysphoria appears to be increasing worldwide [3–5].

Gender dysphoria is generally accompanied by dissatisfaction with physical appearance, and a negative body image has been shown to be more prevalent among transgender women than transgender men [6]. Furthermore, compared to the general population, individuals with gender dysphoria show a higher psychiatric morbidity, which improves following treatment [7–9].

With regards to quality of life, there is a lack of consensus in the field. A few previous studies have indicated that transgender individuals also experience lower quality of life than the general population [10–12], even after gender reassignment surgery [11]. Conversely, other studies have found no difference in quality of life or psychological functioning between transgender individuals and the general population [13–16]. A major shortcoming concerning all previous studies includes a low number of participants, and one previous study has followed patients prospectively with regards to quality of life [17].

## Aims

Is quality of life lower among individuals with gender dysphoria, and how is quality of life affected by gender reassignment therapy? Our aim in this study was to examine and to follow the quality of life of transgender women undergoing gender reassignment surgery.

## Methods

We performed a prospective cohort study on individuals with a diagnosis of gender dysphoria (F64.0 in ICD-10) undergoing male-to-female gender reassignment surgery at Karolinska University Hospital between 2003 and 2015. All patients presenting at the clinic were invited to participate. No exclusion criterion was applied. Patients who denied participation at first visit were not asked again to participate. The quality of life questionnaire was distributed to patients pre-operatively, as well as 1, 3, and 5 years post-operatively.

We used the Swedish version of the self-reported physical and mental health Short Form-36 Health Survey (SF-36), which measures quality of life across eight emotional and physical domains; mental health, vitality, bodily pain, social functioning, role emotional, role physical, physical functioning, and general health, as well as perceived health compared to 1 year previously [18]. The answers on the individual questions are converted using a standardized transformation protocol to scores from 0 to 100 on the eight included dimensions, where 100 represents the highest possible quality of life. The question regarding perceived health compared to 1 year previously is not converted; the replies are on scale 1–5 where 1 is good (“much better now”) and 5 is bad (“much worse now”). The validity of the Swedish SF-36 questionnaire has been thoroughly examined [19–21].

The results were compared between the different measure points, as well as between the study group and the general population. A sub-analysis looking only at the individuals with complete follow-up at all measure points was also performed.

Informed consent was given by all participants and the study was approved by the Stockholm Ethical Board under approval DNR 2005/418–31/3.

### Statistical analysis

Scores on each of the eight dimensions in SF-36 were calculated for each individual, for every measure point, according to the SF-36 interpretation and scoring reference book [18]. Means, standard deviations, and confidence intervals were calculated. We used one-way ANOVA tests to assess the difference between mean scores on the different dimensions. Two-tail *p* values were calculated with a significance level of 0.05. Cronbach’s alpha was used to evaluate internal consistency. All calculations were performed using STATA version 13 (StataCorp 2013 Stata Statistical Software, Collage Station, TX, USA).

## Main outcome measures

Main outcome measures were mean scores on the different dimensions of the SF-36 questionnaire.

## Results

Of 190 individuals included in the study, a majority completed the SF-36 on at least two occasions, and 17 completed the questionnaire on all four measure points. One hundred forty-six individuals completed the questionnaire pre-operatively, 108 at year 1, 64 at year 3, and 43 at year 5. Reasons for loss to follow-up included deceased, moved abroad, and moving without forwarding address and no registration in the Swedish residential register. The mean age of the participants was 36 years (range 19 to 76 years).

Compared to the general population (all ages), transgender women rated their quality of life significantly lower in the dimensions mental health, vitality, social functioning, role emotional, and general health (Table 1). Transgender women rated their bodily pain and physical functioning higher than the general population.

**Table 1:** Scores on SF-36 for individuals in the study compared to the general population [18]

	Year 0 (study pop)		General population (women, all age groups)	
	Mean (SD)	95 % CI	Mean (SD)	95 % CI
Mental health	66.6 (24.2)	62.7–70.6	79.6 (19.4)	79.0–80.2
Vitality	58.8 (25.3)	54.6–62.9	66.7 (23.2)	66.0–67.4
Bodily pain	80.1 (25.3)	75.9–84.3	72.7 (26.5)	71.9–73.4
Social functioning	73.7 (27.0)	69.1–78.2	87.5 (20.8)	86.9–88.1
Role emotional	69.5 (39.7)	62.9–76.0	84.0 (30.9)	83.1–85.0
Role physical	82.5 (30.4)	77.5–87.5	81.6 (33.1)	80.6–82.6
Physical functioning	91.2 (13.7)	89.0–93.4	86.2 (20.4)	85.6–86.8
General health	52.0 (10.4)	50.3–53.7	75.1 (22.7)	74.5–75.8

The mean scores of the eight dimensions at time 0, 1, 3, and 5 are presented in Table 2. Internal consistency was high (Cronbach’s alpha 0.86).

**Table 2:** Scores on SF-36 for all individuals in the study

	0 year		1 year		3 years		5 years	
	Mean (SD)	95 % CI	Mean (SD)	95 % CI	Mean (SD)	95 % CI	Mean (SD)	95 % CI
Mental health	66.6 (24.2)	62.7–70.6	70.1 (24.0)	65.5–74.6	67.7 (25.3)	61.4–73.9	66.1 (26.6)	58.2–74.1
Vitality	58.8 (25.3)	54.6–62.9	61.1 (25.5)	56.2–65.9	59.2 (23.8)	53.3–65.0	57.3 (26.6)	49.4–65.3
Bodily pain	80.1 (25.3)	75.9–84.3	82.1 (24.4)	77.4–86.7	78.6 (28.0)	71.6–85.6	72.5 (26.5)	64.5–80.4
Social functioning	73.7 (27.0)	69.1–78.2	77.5 (27.7)	72.2–82.8	73.8 (28.4)	66.8–80.8	69.8 (29.4)	60.8–78.9
Role emotional	69.5 (39.7)	62.9–76.0	69.1 (41.2)	61.3–76.9	65.1 (41.7)	54.8–75.4	59.7 (44.0)	46.5–72.9
Role physical	82.5 (30.4)	77.5–87.5	82.9 (32.7)	76.7–89.2	79.3 (33.5)	71.1–87.5	70.9 (42.2)	58.3–83.6
Physical functioning	91.2 (13.7)	89.0–93.4	92.4 (13.9)	89.8–95.0	89.7 (17.6)	85.4–94.1	91.5 (11.8)	88.0–95.1
General health	52.0 (10.4)	50.3–53.7	51.9 (12.2)	49.6–54.2	50.0 (12.1)	47.0–53.0	48.1 (12.6)	44.2–51.9

The highest values were attained for post-operative year 1, where mean scores showed a trend to be higher compared to year 0. Compared to year 0, mean scores in year 5 showed a slight trend to be lower for some of the dimensions. None of these trends were statistically significant (*p* > 0.05).

When comparing their current perceived health to that of 1 year previously, the mean responses for years 0, 1, 3, and 5 were 2.4, 2.1, 2.8, and 2.8, respectively. The improved score for year 1 differed significantly from year 0 (*p* < 0.05). The scores for year 5 and 3 were significantly worse compared to year 1 (*p* < 0.0001), but not to year 0.

In a sub-analysis looking only at the 17 individuals with complete follow-up at all measure points, the results were similar.

## Discussion

We performed a large, prospective cohort study of the quality of life of 190 transgender women undergoing gender reassignment surgery. Transgender women reported a lower quality of life, both physical and mental health, than the general population. Although surgical treatment led to an initial trend towards improved quality of life, this decreased with time (up to 5 years).

The finding that quality of life seems to decrease with time, although statistically non-significant, is interesting and underlines the need of prospective studies with long follow-up time. The reasons could be disappointment in long-term effects of surgical treatment, or simply reflect an improvement by treatment from baseline quality of life not sufficient to reach the level of the general population. Another reason could be that only those dissatisfied with treatment, or suffering from complications, completed the follow-up questionnaires at 3 and 5 years and that the results from these measure points do not reflect the true mean of the population under study. The reason for a declining trend in general quality of life after an initial improvement after surgery could also perhaps be explained by the fact that the quality of life in the general population also shows a declining trend with time. Possibly, the improvement resulting from surgical care leads to a higher baseline prior to the patients’ temporal decline in quality of life. This can however not be clearly determined without a control group of non-surgically treated transgender patients. Such a control group would call for a randomized controlled study of a kind that would be an ethical challenge to perform in this patient population.

To our knowledge, very few studies have previously investigated quality of life prospectively, before and after gender reassignment surgery with limitations including low patient numbers, lacking comparison groups, and with limited follow-up time [17]. The participants in a previous study [15], which used a different questionnaire, reported improvement in psychological and social domains after gender reassignment surgery but a worsening in physical health domains. In comparison, our study subjects reported an improvement in almost all dimensions 1 year post-operatively; however, that improvement faded over follow-up time, similar to the time-wise decline of quality of life that is reported by the general population [18].

Our findings on lower quality of life in transgender women compared to the general population of women is in line with some previous studies [10–12] and in contrast to others; however, one of these were performed on transgender men only [14].

Our study has several strengths. Based in Sweden, where affordable care is available to everyone, the participants are well diagnosed at presentation at our clinic. The study design, following a well-defined cohort prospectively, enables an examination of the impact of gender reassignment surgery and time on the quality of life. Furthermore, the SF-36 questionnaire is well validated in several populations [18–21]. Previous studies of SF-36 have shown physical health scales to be strongly associated with age and females in general report poorer health than males [20]. Limitations of the study include incomplete follow-up and the inability to adjust results for clinical factors such as comorbidities, sociodemographic factors, and hormonal treatment. There is no gold standard for measuring quality of life, and so even though the SF-36 questionnaire has been validated in Sweden and elsewhere, it is hard to determine whether SF-36, or any other quality of life measurement tool, is capturing the intended aspect of the individual’s experience. For example, body image is known to be very important among individuals with gender dysphoria and is not necessarily captured entirely by SF-36, and we were not able to measure this separately [6]. Furthermore, the data at 3 and 5 years post-operatively are less reliable since the response rate among patients was significantly decreased compared to earlier time points. However, in the sub-analyses performed on only the individuals with complete follow-up, the results were similar to those of the entire cohort.

The major finding of clinical importance is the poor quality of life reported by transgender women compared to the general population, confirming the vulnerability of this population, and underlining the need for appropriate care and treatment.
